# Supplementary material for: The anti-angiogenic and anti-proliferative activity of n-benzoyl-3-phenyl isoserine derivative containing 1,2,3-triazole and quinoline rings
Source: PLoS One. 2026 May 14;21(5):e0344390. doi: 10.1371/journal.pone.0344390 (PMC13175369; doi:10.1371/journal.pone.0344390)
Supplement: Attachment — Submitted filename: Response to Reviewers.docx [file pone.0344390.s002.docx]

**Response to Reviewers**

We sincerely thank the reviewer and the editorial team for their thoughtful and constructive comments on our manuscript. We truly appreciate the time and effort invested in evaluating our work. The feedback has been invaluable in enhancing the clarity, scientific rigor, and overall quality of the manuscript.

All journal requirements have been fully addressed, including formatting per PLoS ONE style, ORCID ID validation, detailed animal ethics methods, abstract consistency, and Supporting Information captions and citations.

In this revised version, we have carefully addressed each of the reviewer’s points in a systematic manner. Below, we provide detailed responses to each comment, highlighting the revisions and rationale behind them. We hope the improvements meet your expectations and contribute positively to the scientific value of our work.

1. **The abstract should be systematically improved. It is recommended to give a brief background on cancer and the importance of selected target for cancer treatment, then the importance and aim of your work, then the experiments conducted and their outstanding results, then finally write what you conclude from this work.**

We sincerely thank the reviewer for this constructive and insightful suggestion. We greatly appreciate your guidance in helping us improve the structure and clarity of the abstract. In response, we have carefully revised the abstract to follow the recommended structure: starting with a brief background on cancer and the rationale for targeting VEGFR-2, followed by the significance and aim of our study, a concise overview of the conducted experiments and key findings, and concluding with the implications of our results.

The revised abstract:

One of the biggest causes of death globally is cancer, which develops tumors characterized by angiogenesis and excessive cell proliferation. Cancer treatments targeting these pathways, especially those involving vascular endothelial growth factor, have had encouraging results. This study examined the anti-angiogenic and anti-proliferative properties of a phenyl isoserine derivative compound. The ex vivo anti-angiogenic activity was examined by rat aorta ring (RAR) and in vivo chorioallantoic membrane (CAM) assays. The in vitro anti-proliferative properties of the compound were analyzed utilizing Human umbilical vein endothelial (HUVEC), colon cancer, and breast cancer cell lines. Quantitative real-time PCR was used to measure gene expression to evaluate the vascular endothelial growth factor receptor 2 (VEGFR-2) gene expression. An evaluation was conducted on the compound's SwissADME profile, molecular docking, and molecular dynamics simulations against the VEGFR-2 receptor. The phenyl isoserine derivative demonstrated significant anti-angiogenic activity in both the RAR and CAM assays, with a half-maximal inhibitory Concentration (IC50) value of 18.91 μg/mL in the RAR assay. The compound also exhibited anti-proliferative effects against HUVEC, colon cancer, and breast cancer cells, with IC50 values of 68.85, 124.75, and 112.31 μg/mL, respectively. The results of the Real-Time PCR test showed that at a concentration of 200 μg/mL, there was a significant decrease in the expression of the VEGFR-2 gene in Kaposi’s Sarcoma (KS) cells. The compound exhibited membrane permeability and dispersion, as expected based on the SwissADME profile, with a consensus log P o/w value of 3.14, while also indicating potential cytochrome P450 enzyme inhibition and poor water solubility (Log Sw value: -8.34). Molecular docking studies revealed a higher PLP fitness score (91.496) for the compound compared to axitinib (78.748), suggesting a favorable binding to VEGFR-2. However, axitinib demonstrated a more negative binding energy (−14.548 kcal/mol vs. −7.715 kcal/mol), reflecting stronger energetic stabilization under docking conditions. Molecular dynamics simulations showed that legend exhibits stable binding and induces greater structural stabilization of the target protein compared to axitinib, suggesting its potential as a more effective VEGFR-2 inhibitor. The phenyl isoserine derivative was found to have a potent inhibitory effect on angiogenesis and VEGFR-2 gene activity, indicating it could be a promising candidate for anti-cancer medicine.

2. **Many typos and grammatical errors need fixation. The whole manuscript is recommended to be revised by a native English speaker.**

We express our heartfelt gratitude to the reviewer for this insightful observation. The work has been meticulously reviewed to rectify typographical, grammatical, and stylistic mistakes. We have also carefully evaluated the material to enhance clarity and coherence and worked with a native English speaker to ensure the language reaches the required standard. We hope the revised version indicates a considerable improvement in linguistic quality and readability.

3. **Some abbreviations need to be defined for the first time. Please check**

We appreciate the reviewer’s attention to detail. We have carefully reviewed the entire manuscript to ensure that abbreviations are clearly defined upon their first occurrence. Thank you for highlighting this important aspect.

4. **The introduction is too long. It needs to be shorter and more focusing.**

Thank you for this valuable suggestion. Following your recommendation, we have substantially revised the introduction by removing redundant background details and streamlining the content to better focus on the significance of VEGFR-2 as a therapeutic target and the rationale behind our compound design. The revised introduction has been condensed from two pages to one, enhancing clarity and precision.

5. **The authors should** **illustrate the essential pharmacophores of VEGFR-2 inhibitors and some previously reported VEGFR-2 inhibitors** **bear the same scaffold of the derivative under investigation.**

We are grateful for this insightful recommendation. In response, we have now clearly illustrated the essential pharmacophoric features of VEGFR-2 inhibitors in both the Introduction and Discussion sections. Furthermore, we have highlighted how our triazolo-quinoline derivative shares key structural motifs with previously reported VEGFR-2 inhibitors, including hinge-binding heterocycles and hydrophobic scaffolds. Supporting references (e.g., McTigue et al., 2009; and the study on quinoxaline-based VEGFR-2 inhibitors) have been incorporated to substantiate this comparison and reinforce the relevance of our scaffold within this target class.

Part of the introduction:

(VEGFR-2 inhibitors often include a consistent pharmacophore model that includes four critical components: a hinge-binding heteroaromatic ring, a hydrophobic central scaffold, interaction with the aspartate-phenylalanine-glycine (DFG) motif, and a solvent-exposed polar tail. These characteristics facilitate high-affinity and selective inhibition via critical interactions with hinge residues (e.g., Cys919), the hydrophobic pocket, and Asp1046 within the DFG motif. [4]. Many FDA-approved inhibitors of the VEGFR-2 enzyme (axitinib, cabozantinib, lenvatinib, sorafenib, and tivozanib) possess four critical pharmacophoric characteristics: a hinge-binding heteroaromatic ring, a central aromatic linker, a pharmacophoric hydrogen bond donor/acceptor moiety interacting with the DFG motif, and a terminal lipophilic group that occupies the allosteric pocket. Previous studies have effectively integrated these characteristics into quinoxaline-based frameworks, showcasing their promise as VEGFR-2-targeted anticancer drugs [5].

Part of the discussion:

(The triazolo-quinoline scaffold in the investigated N2 compound mirrors established pharmacophoric features of VEGFR-2 inhibitors, including hinge-binding via the quinoline ring and potential aspartate-phenylalanine-glycine (DFG) motif interactions via the triazole moiety. Structurally, this is comparable to known inhibitors like axitinib and sorafenib, supporting its rational design as a VEGFR-2-targeted anticancer agent [4]. The N2 compound investigated in this study shares critical pharmacophoric elements with reported VEGFR-2 inhibitors, such as the hinge-binding quinoline ring and hydrophobic and H-bonding moieties analogous to those in quinoxaline derivatives [5]. These structural similarities support the rational design of N2 as a VEGFR-2 inhibitor and are consistent with previously validated scaffolds showing promising anticancer and VEGFR-2 inhibitory activity.)

References:

4. McTigue M, Murray BW, Chen JH, Deng Y-L, Solowiej J, Kania RS. Molecular conformations, interactions, and properties associated with drug efficiency and clinical performance among VEGFR TK inhibitors. Proceedings of the National Academy of Sciences 2012;109:18281–9. https://doi.org/10.1073/pnas.1207759109.

5.	Alanazi MM, Elkady H, Alsaif NA, Obaidullah AJ, Alanazi WA, Al-Hossaini AM, et al. Discovery of new quinoxaline-based derivatives as anticancer agents and potent VEGFR-2 inhibitors: Design, synthesis, and in silico study. J Mol Struct 2022;1253:132220. https://doi.org/10.1016/j.molstruc.2021.132220.

6. **It is highly recommended to conduct molecular docking using PDB codes that include triazolo quinoline derivatives as co-crystallized ligand to get more reliable results.**

We express our heartfelt gratitude to the reviewer for this invaluable recommendation. We comprehensively searched the Protein Data Bank (PDB) for VEGFR-2 crystal structures co-crystallized with triazolo-quinoline derivatives. Regrettably, to our knowledge, no such constructions are presently accessible. Notwithstanding this constraint, we employed the VEGFR-2 structure (PDB: 5EW3), a validated kinase domain frequently utilized in analogous investigations, and conducted molecular docking using GOLD software with the PLP scoring tool to evaluate our drug's binding affinity and conformation. We utilized the Glide software from the Schrödinger suite to evaluate our drug's binding energy. Additionally, to enhance our docking outcomes, we performed molecular dynamics (MD) simulations to assess the stability of the docked complex over time. We trust that these initiatives demonstrate our commitment to providing thorough and reliable binding interaction evaluations, despite the unavailability of the specified co-crystallized ligand.

Results: the Glide software from the Schrödinger suite to evaluate our drug's binding energy

**The 2D and 3D interaction maps of the N2 compound and Axitinib with receptor (5EW3) of vascular endothelial growth factor receptor 2 (VEGFR-2)**

|  | **2D interaction map** | **3D interaction map** | **Binding energy (kcal/mol)** |
| --- | --- | --- | --- |
| **N2 compound** |  |  | **-14.548** |
| **Axitinib** |  |  | **-7.715** |

7. **All** **biological investigations should be carried out using positive controls.**

We sincerely thank the reviewer for this critical observation. Following standard experimental protocols, we employed bevacizumab as a positive control in our biological studies, which included the rat aortic ring assay, CAM assay, and VEGF gene expression study. Bevacizumab was chosen for its clinically established function as an anti-angiogenic drug that targets VEGF. Including this reference ensured our experimental results' reliability and interpretability. We recognize the necessity of explicitly articulating this in the manuscript and have revised the pertinent portions accordingly. We apologize for the delay in our response, which was necessitated by the time required to provide the positive control with the necessary care and precision.

Results of the use of **Bevacizumab as a positive control** in biological investigations

|   **100 μg/ml Bevacizumab (positive control) on blood vessel formation in rat aorta rings** |   **Anti-angiogenesis activity of the different concentrations of the N2 compound along with Bevacizumab (positive control) and 1% DMSO (negative control) in ex vivo aortic ring model** |
| --- | --- |
|   **The degree of the inhibition zone of blood vessel growth in vivo (CAM) assay:** Bevacizumab (positive control) |   **Fold change in gene expression of the VEGF gene after exposure to different concentrations of the N2 compound, determined using the ΔΔCt method.** Bevacizumab (positive control) |

8. **Why authors have selected axitinib, in particular, as a reference drug for molecular docking study.**

Axitinib was selected as a reference compound due to its well-established and FDA-approved role as a selective VEGFR-2 inhibitor used clinically in anti-angiogenic cancer therapy. Structurally, axitinib binds with high affinity to the ATP-binding site of VEGFR-2 and serves as a benchmark in molecular docking studies evaluating novel VEGFR-2-targeting agents. Its crystallographic data are readily available (PDB: 5EW3), enabling accurate comparison of binding interactions and scoring against our test compound. We have clarified this rationale in the revised manuscript to reflect the scientific justification behind our choice better.

9. **The** **molecular docking program validation should be carried out.**

We sincerely thank the reviewer for raising this important point. In response, we performed rigorous validation of our molecular docking protocol. Initially, we conducted a re-docking of the co-crystallized ligand into the VEGFR-2 active site (PDB ID: 5EW3), and the resulting Root Mean Square Deviation (RMSD) value between the docked pose and the native conformation was below 2.0 Å, indicating good reproducibility and validating the docking accuracy. To strengthen our findings, we also used Glide software (Schrödinger suite) to evaluate our compound's binding energy and docking interactions, providing an additional layer of precision and cross-platform consistency. Moreover, to simulate fundamental physiological dynamics and validate the stability of ligand-receptor interactions, we performed Molecular Dynamics (MD) simulations, which included:

Root Mean Square Deviation (RMSD)

Radius of Gyration (Rg)

Root Mean Square Fluctuation (RMSF)

Solvent-Accessible Surface Area (SASA)

Hydrogen bond analysis

Principal Component Analysis (PCA)

These simulations demonstrated that our compound maintained a stable interaction within the VEGFR-2 binding site throughout the simulation, supporting the reliability of our docking results. All relevant figures and data are provided in the revised manuscript, and the next point.

10. **It is highly recommended to conduct**

We sincerely thank the reviewer for this valuable suggestion. In response, molecular dynamics (MD) simulations were successfully performed for the VEGFR-2 receptor (PDB ID: 5EW3) in complex with both the N2 compound and the reference drug, as well as for the unbound receptor. The simulations used GROMACS 2023.0 on a Linux 22.4 system, employing the TIP3P water model for solvation.

To comprehensively evaluate the stability and dynamic behavior of the complexes, we calculated key metrics including Root Mean Square Deviation (RMSD), Radius of Gyration (Rg), Root Mean Square Fluctuation (RMSF), Solvent-Accessible Surface Area (SASA), Hydrogen bonding, and Principal Component Analysis (PCA). These analyses confirmed the structural stability and meaningful interaction of the N2 compound with the receptor, comparable to the reference drug. The full results are now included in the revised manuscript.

Results of Molecular Dynamics simulations:

|   A |   B |
| --- | --- |
| **Interaction Analysis: (a) The first frame and last frame interaction of (5EW3) with Axitinib. (b) The first frame and last frame interaction of (5EW3) with N2** | |
|   a |   b |
| **(a) principal component analysis of three states (protein only(5EW3)) with Axitinib, N2 compound. (b) comparison of PCA variance of unliganded protein(5EW3) and liganded (protein-ligand (N2 compound), protein-Axitinib)** | |
| **SASA Comparison: Protein Only (5EW3), Axitinib and N2 compound** |   **RMSD Comparison: Protein Only (5EW3), Axitinib and N2 compound** |
| **Rg Comparison: Protein Only(5EW3), Axitinib and N2 compound.** | **Root Mean Square Fluctuation (RMSF) Analysis Reveals Enhanced Structural Stability of the Protein(5EW3) upon Binding with N2 Compound Compared to Axitinib** |
|   **Hydrogen Bonds Between the Protein(5EW3) and Axitinib, N2 compound** | |

We sincerely appreciate the constructive comments and valuable suggestions provided by the reviewer, which have significantly helped us improve the quality and clarity of our manuscript. We have meticulously addressed each point and amended the manuscript accordingly. We believe these changes have strengthened the scientific rigor and presentation of our study. We hope that the revised version meets the journal’s standards and the reviewer’s expectations.

Best Regards
