# Supplementary material for: The anti-angiogenic and anti-proliferative activity of n-benzoyl-3-phenyl isoserine derivative containing 1,2,3-triazole and quinoline rings
Source: PLoS One. 2026 May 14;21(5):e0344390. doi: 10.1371/journal.pone.0344390 (PMC13175369; doi:10.1371/journal.pone.0344390)
Supplement: Attachment — Submitted filename: Response_to_Reviewers_auresp_2.docx [file pone.0344390.s003.docx]

**Response to Reviewers**

We appreciate the Editor and Reviewers for their constructive and insightful comments, which have enhanced the quality and clarity of our manuscript. The delay in submitting the revised version is due to the time required to regenerate and acquire high-resolution figures, especially those generated by molecular dynamics simulation software, to ensure accuracy and presentation quality. All comments have been systematically addressed as outlined below.

**1]** The manuscript uses multiple names: “N2 compound”, “phenyl isoserine derivative”, “N-Benzoyl-3-phenyl isoserine derivative”, etc. Choose one short label (e.g., “N2”) and use it consistently after the first full chemical name. Ensure the first instance includes the full IUPAC name and a 2D structure (Fig. 1).

We appreciate the reviewer's constructive feedback. We have amended the manuscript to consistently use a uniform label (“N2”). The whole IUPAC nomenclature and chemical structure of N2 are included in Fig. 1 as requested.

(N2: 3-benzamido-3-phenyl-2-(4-((quinoline-8-yloxy) (methyl))-1H- 1,2,3-triazole-1-yl) propanoic acid) (N-Benzoyl-3-phenyl isoserine derivative containing 1,2,3-triazole and quinoline rings).

**2]** The manuscript alternates between “VEGF gene”, “VEGFR-2 gene”, and “VEGF” in ways that may confuse readers. Use standard nomenclature (e.g., VEGFR-2 for the receptor; VEGF-A for the ligand if measured). Italicize gene symbols where journal style requires it and be consistent throughout.

We thank the reviewer for this helpful comment. We have revised the manuscript to use a **consistent and standard nomenclature** throughout. Specifically:

- All mentions of the receptor now use VEGFR-2 (italicized as per journal style).
- All previous inconsistencies in gene/receptor naming have been corrected to ensure clarity and readability.

**3]** Some abbreviations (e.g., RAR, CAM, HUVEC, MD, RMSD) are used before being defined. Define each abbreviation at first mention and consider adding an abbreviations list.

We appreciate the reviewer's insightful comment. All abbreviations, including RAR, CAM, HUVEC, MD, and RMSD, have been defined upon their initial occurrence in the manuscript. A comprehensive **list of abbreviations** has been included to enhance clarity and readability.

**abbreviations list:**

RAR: rat aorta ring, CAM: chorioallantoic membrane, HUVEC: Human umbilical vein endothelial, RT-qPCR: real-time quantitative polymerase chain reaction, *VEGFR-2*: vascular endothelial growth factor receptor 2, IC_50_: half-maximal inhibitory Concentration, KS: Kaposi’s Sarcoma, PLP: Precise Linear Potential, MD: Molecular docking, MCF-7: breast cancer cell lines, ADME: absorption, distribution, metabolism, and excretion, HCT-116: colon cancer cell lines, DMSO: Dimethyl sulfoxide, SD: standard deviation, PDB: Protein Data Bank, PCA: principal component analysis, SASA: Solvent-Accessible Surface Area, RMSD: Root Mean Square Deviation, ROG: Radius of Gyration, RMSF: Root Mean Square Fluctuation and N2: N-Benzoyl-3-phenyl isoserine derivative containing 1,2,3-triazole and quinoline rings

**4]** Minor typos and awkward phrases remain (e.g., “legend exhibits stable binding”, probably “ligand”). Fix obvious typos and awkward sentences; ensure subject/verb agreement. A quick pass by a native English editor or use of proofreading software would resolve these.

We appreciate the reviewer’s observation. All typographical errors and awkward phrases have been carefully revised, including the correction of “legend” to “ligand.” We also reviewed the manuscript to ensure proper subject–verb agreement and overall clarity of language. A thorough proofreading pass has been completed to improve readability.

**5]** Some panels are low resolution and legends lack detail (e.g., exact n, statistical test used, meaning of error bars). Upload high-resolution figures, label panels (A, B, C), and update each legend to include sample size (n), test used, definition of error bars (SD or SEM), and exact p-value formatting.

The reviewer’s comment is appreciated. All figures have been substituted with high-resolution versions, and each panel has been distinctly labeled (a-c) to improve readability. Additionally, all figure legends have been revised to incorporate comprehensive details regarding sample size (n), the statistical tests utilized, the definition of error bars (SD or SEM), and consistent p-value formatting. The revisions enhance the clarity and scientific transparency of the figures considerably.

**Fig. 3.** **Dose-response relationship for the N2: (a)** Anti-angiogenesis activity of the differentN2 (12.5 – 200 µg/mL) (Mean ± SD percentage of blood vessels inhibition) of the N2 along with Bevacizumab (positive control) and 1% DMSO (negative control) using ANOVA post hoc (Tukey’s test). Different letters (a, b, c, d, e, f) were considered significantly different at p < 0.05 in *ex vivo* aortic ring assay.

**Fig. 7. Mean ± SD fold of *VEGFR-2*** **gene after exposure to different N2 concentrations (12.5 – 200 µg/mL) compared to Bevacizumab (positive vehicle) treatment.** Measurements of untreated cells were normalized to 1.0-fold expression and considered the control. Comparison between different groups was achieved using ANOVA post hoc (Tukey’s test) and different letters (a, b, c) considered significantly different at p < 0.05, determined by the ΔΔCt method.

(a)

(b)

**Fig. 9: (a) The** **first frame and** **last frame interaction of (5EW3) with Axitinib. (b) The first frame and last frame interaction of (5EW3) with N2**

(a) (1): PCA-Protein	(2) PCA-axitinib	(3) N2

| (b) PCA variance principal component | (C) PCA variance explained |
| --- | --- |

**Fig. 10: (a) principal component analysis of three states (protein only(5EW3)) with Axitinib, N2. (b) comparison of PCA variance of unliganded protein(5EW3) and liganded (protein-ligand (N2), protein-Axitinib)**

(a) **Protein	(b) Axitinib	(c) N2**

**Fig. 11: SASA Comparison: Protein Only (5EW3), Axitinib, and N2**

(a) **Protein (5EW3)	(b) Protein (5EW3) and Axitinib	(c) Protein (5EW3) and N2**

**Fig. 12: RMSD Comparison: Protein Only (5EW3), Axitinib and N2**

**Fig. 13: Rg Comparison: Protein Only(5EW3), Axitinib and N2.**

**Fig. 14: Root Mean Square Fluctuation (RMSF) Analysis Reveals Enhanced Structural Stability of the Protein(5EW3) upon Binding with N2 Compared to Axitinib**

**Fig. 15: Hydrogen Bonds Between the Protein(5EW3) and Axitinib, N2**

**6]** The manuscript uses “P ≤ 0.05” and “P < 0.05” inconsistently and often gives only threshold statements. Use consistent lowercase or uppercase p per journal style (commonly “p”) and report exact p-values where feasible (e.g., p = 0.0001), and always state which test was used in figure legends.

We value the reviewer’s insightful feedback. The paper has been meticulously edited to guarantee uniformity in p-value formatting. All occurrences of “P ≤ 0.05” and “P < 0.05” have been standardized to the journal's preferred format (“p < …”). Exact p-values have been supplied where applicable, rather than threshold assertions.

Furthermore, all figure legends have been revised to incorporate: sample size (n), the statistical test employed, the definition of error bars (SD or SEM), and the associated p-values.

**7]** pH was shown as “7.5” and elsewhere as “7.5,” decimal reporting varies. Standardize number formatting (e.g., one or two decimal places consistently) and ensure percent signs have spaces.

We appreciate the reviewer's insightful observation. Numerical formatting in the manuscript has been standardized. Decimal places have been standardized (e.g., pH consistently reported as “7.5”), and percentage values adhere to a uniform format. The manuscript, along with tables and figure legends, has been revised to maintain consistency in numerical presentation.
